# Organization of Subunits in the Membrane Domain of the Bovine F-ATPase Revealed by Covalent Cross-linking*

**DOI:** 10.1074/jbc.M115.645283

**Published:** 2015-04-07

**Authors:** Jennifer Lee, ShuJing Ding, Thomas B. Walpole, Andrew N. Holding, Martin G. Montgomery, Ian M. Fearnley, John E. Walker

**Affiliations:** From the ‡ The Medical Research Council Mitochondrial Biology Unit, Cambridge Biomedical Campus, Hills Road, Cambridge CB2 0XY, United Kingdom and; § The Medical Research Council Laboratory of Molecular Biology, Cambridge Biomedical Campus, Francis Crick Avenue, Cambridge CB2 0QH, United Kingdom

**Keywords:** ATP synthase, membrane energetics, mitochondria, protein structure, protein-protein interaction, supernumerary membrane subunits, topography

## Abstract

The F-ATPase in bovine mitochondria is a membrane-bound complex of about 30 subunits of 18 different kinds. Currently, ∼85% of its structure is known. The enzyme has a membrane extrinsic catalytic domain, and a membrane intrinsic domain where the turning of the enzyme's rotor is generated from the transmembrane proton-motive force. The domains are linked by central and peripheral stalks. The central stalk and a hydrophobic ring of c-subunits in the membrane domain constitute the enzyme's rotor. The external surface of the catalytic domain and membrane subunit a are linked by the peripheral stalk, holding them static relative to the rotor. The membrane domain contains six additional subunits named ATP8, e, f, g, DAPIT (diabetes-associated protein in insulin-sensitive tissues), and 6.8PL (6.8-kDa proteolipid), each with a single predicted transmembrane α-helix, but their orientation and topography are unknown. Mutations in ATP8 uncouple the enzyme and interfere with its assembly, but its roles and the roles of the other five subunits are largely unknown. We have reacted accessible amino groups in the enzyme with bifunctional cross-linking agents and identified the linked residues. Cross-links involving the supernumerary subunits, where the structures are not known, show that the C terminus of ATP8 extends ∼70 Å from the membrane into the peripheral stalk and that the N termini of the other supernumerary subunits are on the same side of the membrane, probably in the mitochondrial matrix. These experiments contribute significantly toward building up a complete structural picture of the F-ATPase.

## Teaser

**Background:** Bovine mitochondrial F-ATPase has six uncharacterized membrane subunits.

**Results:** Labeled lysines have been mapped onto its incomplete structure.

**Conclusion:** Subunit ATP8 extends about 70 Å from the membrane into the peripheral stalk. The N-terminal regions of five others are in the mitochondrial matrix.

**Significance:** Subunit ATP8 is in the stator. Five other subunits form a subdomain.

## Introduction

The F-ATPase from bovine mitochondria is a membrane-bound protein assembly of ∼30 polypeptides of 18 different kinds with a combined molecular mass of ∼650 kDa (1, 2). The enzyme has a membrane extrinsic globular F_1_-catalytic domain that is attached to the membrane domain by central and peripheral stalks. The F_1_ domain is an assembly of three α-subunits and three β-subunits arranged in alternation in a spherical complex around an elongated α-helical structure in the γ-subunit (3), and the three catalytic sites of the enzyme lie at interfaces between the α- and β-subunits. The γ-subunit extends from the α_3_β_3_-spherical structure to the membrane domain, where it is augmented by the δ- and ϵ-subunits in the contact region or “foot” with the membrane domain. Together, the γ-, δ-, and ϵ-subunits constitute the central stalk, and the foot makes extensive contacts with a hydrophobic ring of eight c-subunits in the membrane domain of the enzyme (4). The c_8_-ring and the central stalk constitute the rotor of the enzyme. Their rotation as an ensemble at ∼100 Hz carries energy into the catalytic sites of the enzyme, and the turning of the rotor brings about conformational changes in the three catalytic sites in the F_1_ domain that lead to the binding of substrates and the formation of ATP and its release into the matrix. The region of contact between the rotating c-ring and subunit a, another membrane protein of unknown structure, provides the transmembrane pathway for protons to pass from the intermembrane space into the mitochondrial matrix (5), allowing energy from the transmembrane proton-motive force generated by respiration to drive the turning of the rotor. Subunit a forms part of the stator and is in contact with the membrane domain of subunit b, which extends through the peripheral stalk, an elongated subcomplex of single copies of subunits b, d, F_6_, and oscp (6–9) to the “top” of the α_3_β_3_ domain (10). Thus, the stator consists of the α_3_β_3_ domain, plus subunits oscp, b, d, F_6_, and a, and it remains static relative to the turning of the rotor. Atomic resolution structures have been established for the F_1_ c-ring (4) and peripheral stalk domains (7, 8), and a mosaic structure of the enzyme has been built within the envelope of a low resolution structure of the entire complex determined by electron cryo-microscopy (11).

Also associated with the membrane domain of the bovine enzyme are six small proteins, ATP8, e, f, g, DAPIT^3^ (diabetes associated protein in insulin sensitive tissues), and 6.8 kDa proteolipid (6.8PL), with molecular masses in the range 6.3–11.4 kDa (1, 2, 12–14). Each has a single predicted transmembrane α-helix, and based largely on their staining intensities in gel analyses of the subunit composition of the enzyme, it is assumed that there is one copy of each protein per F-ATPase complex, but there are no definitive quantitative data to support this assumption. These proteins are usually referred to as the “supernumerary” subunits as there are no orthologs in bacterial F-ATPases, and they appear not to be involved directly in the synthesis of ATP. In the yeast enzyme the orthologs of subunits e and g are associated with the formation of dimers of the F-ATPase (15, 16), and they probably play the same role in the mammalian enzyme.

In this study the identification of covalent cross-links introduced into the subunits of the bovine F-ATPase by reaction of exposed amino groups with isotopically labeled bifunctional reagents has been employed as a means of gaining information about the orientation and location of the supernumerary subunits within the bovine enzyme complex.

## Experimental Procedures

### 

#### 

##### Analytical Methods

Protein concentrations were estimated by the bicinchoninic acid assay (17). Protein compositions were analyzed by SDS-PAGE in 12–22% or 4–20% polyacrylamide gradient gels or by blue native PAGE (18, 19). Proteins were detected by staining with Coomassie Blue dye.

##### Purification of Monomeric Bovine F-ATPase

Phosphate-washed bovine mitochondrial membranes (10 mg/ml) were extracted with buffer containing 1% (w/v) *n*-dodecyl-β-d-maltoside and 20 mm Tris·HCl, pH 7.3, 10% glycerol (v/v), 0.15 m sodium chloride, 20 mm EDTA, 1 mm tris(2-carboxyethyl)phosphine, 1-palmitoyl-2-oleoyl-*sn*-glycerophosphocholine (100 μg/ml), 1-palmitoyl-2-oleoyl-*sn*-glycerophosphoethanolamine (33 μg/ml), and 1-palmitoyl-2-oleoyl-*sn*-glycero-phospho-(1′-rac-glycerol) (33 μg/ml). The ATP hydrolytic activity of the F-ATPase, measured by an enzyme-coupled assay (20), was inhibited with a recombinant protein consisting of residues 1–60 of the natural bovine inhibitor protein, IF_1_, with a C-terminal glutathione *S*-transferase domain plus hexahistidine, and the active enzyme complex was purified as described before (21) except that the detergent in the buffers, *n*-dodecyl-β-d-maltoside, was replaced by 0.05% (w/v) *n*-dodecyl-β-d-maltose-neopentyl glycol. The ATP hydrolytic activity of the purified enzyme was 50–90 μmol of ATP hydrolyzed/min/mg, and the sensitivity of this activity to inhibition by 0.01% (w/v) oligomycin was 95–99%, indicating that the enzyme was almost entirely coupled in its F_o_ domain. Purified F-ATPase was dialyzed against buffer consisting of 20 mm HEPES, pH 7.3, 0.05% *n*-dodecyl-β-d-maltose-neopentyl glycol (w/v), 10% glycerol (v/v), 50 mm sodium chloride, 1 mm tris(2-carboxyethyl)phosphine, 2 mm magnesium sulfate, 2 mm ATP, 1-palmitoyl-2-oleoyl-*sn*-glycero-phosphocholine (100 μg/ml), 1-palmitoyl-2-oleoyl-*sn*-glycerophosphoethanolamine (33 μg/ml), 1-palmitoyl-2-oleoyl-*sn*-glycerophospho-(1′-rac-glycerol (33 μg/ml).

##### Chemical Cross-linking

**FIGURE 1.**
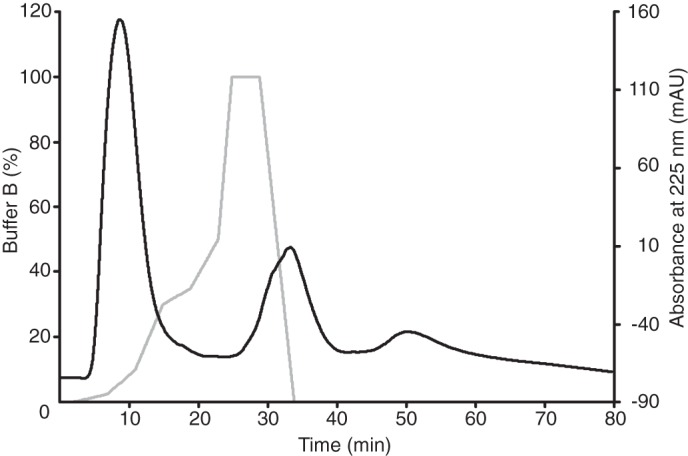
**Fractionation of a tryptic digest of bovine F-ATPase cross-linked with DSS(*d*_0_*d*_12_).** The digest was injected into a column of PolySULFOethyl A equilibrated at a flow rate of 50 μl/min in buffer, pH 2.7, containing 10% acetonitrile and eluted with a gradient of potassium chloride (*gray line*) from 0 to 1.0 m. The absorbance (*mAU*) of the effluent was monitored at 225 nm (*black line*). The cross-linked peptides were identified in fractions collected across the second peak eluting at 27–40 min.

Four deuterium-labeled cross-linking agents were employed. Disuccinimidyl suberate (DSS) and disuccinimidyl glutarate (DSG) are membrane-permeable, whereas di(sulfosuccinimidyl) glutarate (DSSG) and bi(sulfosuccinimidyl) suberate (BS^3^) are membrane impermeable. In the isotope-labeled versions, DSS(*d*_0_/*d*_12_), BS^3^(*d*_0_/*d*_12_), DSG(*d*_0_/*d*_6_), and DSSG(*d*_0_/*d*_6_), (Creative Molecules), the numbers of deuterium atoms, *d*, in the two acyl arms of each reagent are indicated by the subscripted numerals and were supplied and used at molar ratios of the two isotopic forms of 1:1 (22, 23). Freshly purified bovine F-ATPase was diluted to a concentration of 1.22 mg/ml in buffer consisting of 20 mm HEPES, pH 7.3, 0.05% *n*-dodecyl-β-d-maltose-neopentyl glycol (w/v), 10% glycerol (v/v), 50 mm sodium chloride, 1 mm tris(2-carboxyethyl)phosphine, 2 mm magnesium sulfate, 2 mm ATP, 1-palmitoyl-2-oleoyl-*sn*-glycero-phosphocholine (100 μg/ml), 1-palmitoyl-2-oleoyl-*sn*-glycerophosphoethanolamine (33 μg/ml), and 1-palmitoyl-2-oleoyl-*sn*-glycero-phospho-(1′-rac-glycerol (33 μg/ml). The reaction conditions were optimized with DSS(*d*_0_/*d*_12_) dissolved in dimethyl sulfoxide added at concentrations of 0.1–5 mm. The reactions were carried out at pH values of 6.8, 7.3, and 8.0 for 2, 4, or 20 h and at temperatures of 20 or 37 °C. The optimal cross-linking conditions were 2 μm F-ATPase (1.16 mg/ml) at pH 8.0 reacted with 2 mm DSS(*d*_0_/*d*_12_) for 2 h at 20 °C. BS^3^(*d*_0_/*d*_12_), DSG(*d*_0_/*d*_6_) and DSSG(*d*_0_/*d*_6_) were reacted with the enzyme at the same concentration and under the same conditions. The reactions were terminated by the addition of ammonium bicarbonate to a final concentration of 200 mm. The solutions were kept at 20 °C for 30 min. Insoluble material was removed by centrifugation (10,000 × *g*, 5 min, 20 °C), and the supernatants were analyzed by SDS-PAGE and blue native-PAGE. The cross-linked F-ATPases were precipitated with ethanol for 18 h at −20 °C, and the precipitates were recovered by centrifugation and redissolved in 6 m guanidinium hydrochloride in 20 mm Tris-HCl buffer. The proteins were reduced with tris(2-carboxyethyl)phosphine, alkylated with iodoacetamide (10 mm), and re-precipitated. These samples of *S*-alkylated cross-linked F-ATPases were analyzed in three different ways. First, the precipitates were dissolved in 100 mm ammonium bicarbonate and digested at 37 °C for 16 h with trypsin (trypsin:protein, 1:100, w:w). Portions of the digests were diluted with 0.1% formic acid for mass spectrometric analysis. Second, other samples of *S*-alkylated and cross-linked F-ATPase were fractioned by SDS-PAGE, and gel bands or gel regions were excised and digested with trypsin (24). Third, an *S*-alkylated sample of F-ATPase cross-linked with DSS(*d*_0_/*d*_12_) was dialyzed for 18 h against 100 mm ammonium bicarbonate and digested at 37 °C with trypsin (F-ATPase: trypsin, 100:1, w:w). The digest was dried and fractionated on a column of PolySULFOethyl A (50 mm long × 1.0 mm inner diameter; 300 Å pore size; PolyLC, Columbia, MD) equilibrated at a flow rate of 50 μl/min in 10 mm potassium phosphate buffer, pH 2.7, containing 10% acetonitrile. Peptides were eluted with a gradient of 0–1 m potassium chloride in 20 mm potassium phosphate, pH 3.0, containing 10% acetonitrile (see Fig. 1). Fractions were dried and dissolved in 0.3% trifluoroacetic acid, and salts were removed from the samples by passage through a reverse-phase C_18_ ZipTip (Millipore).

##### Analysis of Cross-linked Peptides by Mass Spectrometry

Tryptic digests of protein bands from SDS-PAGE gels were analyzed in a MALDI-TOF-TOF mass spectrometer (Model 4800, ABSciex, Warrington, WA1 1RX, UK) with α-cyano-4-hydroxycinnamic acid as matrix. Peptides were fragmented by collision-induced dissociation with air at a collision energy of 1 kV. Trypsin digests of cross-linked F-ATPases (∼400 fmol), and fractions of peptides from similar digests that had been obtained by cation exchange chromatography were injected into a nano-scale C_18_ reverse phase column. The eluate was introduced directly into the nanospray interface of a LTQ Orbitrap XL ETD mass spectrometer (Thermo-Fisher Scientific, Hemel Hempstead, UK), operated in data-dependent acquisition mode. Up to 10 of the most abundant precursor ions of known charge states, but not singly charged, were selected and fragmented by collision-induced dissociation. The *m*/*z* values of precursor and fragment ions were measured simultaneously in the Orbitrap and ion-trap analyzers, respectively.

##### Data Analysis

Data files were processed with Proteome Discoverer (Thermo-Fisher Scientific). The coverage of the sequences of the subunits of the F-ATPase by the tryptic peptides was determined by comparison of the sequences of peptides with the mammalian subsection of the non-redundant database of the National Center for Biotechnology Information with MASCOT (25). “Mono-linked” peptides, where only one arm of the bifunctional cross-linking agent reacted with a protein amino group, were identified by comparison of the data with a FASTA database containing the sequences of the subunits of the enzyme. In these comparisons the light and heavy isotopes of the hydrolysis and aminolysis derivatives of mono-linked α-amino and ϵ-amino groups, oxidized methionines, and *S*-propionamido- and *S*-carbamidomethyl derivatives of cysteine were considered as possible modifications with the following parameters: peptide mass tolerance, ± 5ppm; fragment mass tolerance, ±0.5 Da; maximum missed cleavages, 2; peptide confidence, at least medium.

##### Identification of Cross-linked and Loop-linked Peptides

Doublet peaks corresponding to the light and heavy isotopically labeled cross-linked peptides (including loop-linked peptides) were identified from their mass differences with Hekate (26). For the doublets for DSS(*d*_0_/*d*_12_) and BS^3^(*d*_0_/*d*_12_) the mass difference was 12.0741 ± 0.0375, and for DSG(*d*_0_/*d*_6_) and DSSG(*d*_0_/*d*_6_) it was 6.0370 ± 0.0375 Da. No limits were imposed on the intensity ratios of the doublet peaks. To be assigned as an isotopic doublet, peptide ions containing light and heavy isotopes were required to elute within 30 s of each other. The mass threshold for matching the masses of the light isotope peak of cross-linked peptides with their calculated theoretical values was 2 ppm. The accuracy threshold used in the annotation of fragment ions was ±0.5 Da. The identified cross-links were ranked according to a modified version of Andromeda (26, 27) with fragment ion data from both the light-and heavy-labeled cross-linked peptides.

Mass spectra from cross-linked and loop-linked peptides with a score of >400 were inspected manually. A cross-link was accepted as being significant if both cross-linked peptides had more than five unique fragment ions assigned to them or if the cross-linked peptide had three-five unique fragments ions assigned to one component and more than five to the other. Accepted loop links had three or more unique assigned fragments. Twenty mono-links and six loop links were detected (data not shown). Peptides were discarded when the majority of high intensity fragment ions were not annotated with a theoretical fragment ion from the associated peptide or when fewer than three fragment ions of a peptide component of a cross-link were detected.

##### Localization of Cross-links in the Structure of the Bovine F-ATPase

A structure of the bovine F_1_-c_8_-peripheral stalk complex was generated from the structures of the bovine F_1_-c_8_-ring (PDB code 2XND (4)), the F_1_-peripheral stalk (PDB code 2WSS (7)), and the peripheral stalk (PDB code 2CLY (8)), and the position of the peripheral stalk was adjusted according a low resolution structure of the intact complex (11). The distances between Cα atoms of lysine residues in the model of the bovine F_1_-c_8_-peripheral stalk complex were estimated with PyMOL and Coot (28, 29). For DSS(*d*_0_/*d*_12_) and BS^3^(*d*_0_/*d*_12_), the maximum permitted inter-Cα distance between connected lysines is 27.4 Å (the 11.4 Å spacer arm plus the length of two lysine side chains, each 6.5 Å, plus twice the coordinate error of 1.5 Å for mobile surface residues). For DSG(*d*_0_/*d*_6_) and DSSG(*d*_0_/*d*_6_) with spacer arms of 7.7 Å, the maximum inter-Cα distance is 23.7 Å.

##### Analysis of Sequences of F-ATPases

Sequences of subunits of F-ATPases were aligned with ClustalW (30). Transmembrane α-helices were predicted with HMMTOP (31), and the secondary structures of membrane extrinsic regions were predicted with PSIPRED (32).

## Results

### 

#### 

##### Optimization of Reaction Conditions for Cross-linking the F-ATPase

**FIGURE 2.**
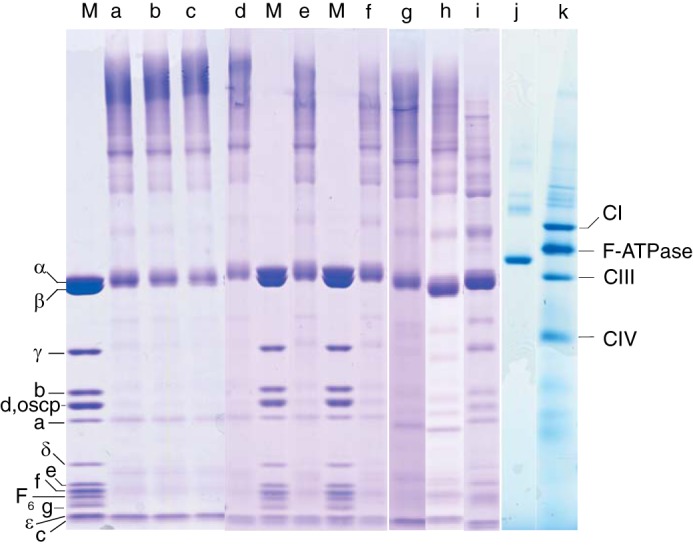
**Effect of varying the reaction conditions on the cross-linking of the bovine F-ATPase.** The enzyme (1.16 mg/ml) was reacted at 37 °C with DSS(*d*_0_/*d*_12_) (*lanes a–f*) or BS^3^(*d*_0_/*d*_12_), DSG(*d*_0_/*d*_6_), or DSSG(*d*_0_/*d*_6_), (*lanes g–i*, respectively) at final reagent concentrations of 2 mm. For reference, the molecular weights of subunits α, oscp, and ϵ are 55,200, 20,900, and 5,600, respectively. The samples of modified enzyme were analyzed by SDS-PAGE (*lanes a–i*) and by blue native PAGE (*lanes j* and *k*). *M* denotes unreacted enzyme; *lanes a–c*, enzyme reacted with DSS(*d*_0_/*d*_12_) for 2, 4, and 20 h; *lanes d–f*, enzyme reacted with DSS(*d*_0_/*d*_12_) for 2 h at pH values of 8.0, 7.3, and 6.8; *lanes g–i*, enzyme reacted for 2 h at pH 8.0 with BS^3^(*d*_0_/*d*_12_), DSG(*d*_0_/*d*_6_), or DSSG(*d*_0_/*d*_6_), respectively; *lane j*, enzyme reacted with DSS(*d*_0_/*d*_12_) for 2 h at pH 8.0; *lane k*, bovine inner mitochrondrial membranes solubilized in the presence of *n*-dodecyl-β-d-maltose-neopentyl glycol. The migration positions of unmodified subunits of the enzyme are indicated on the *left* and on the right of native respiratory complexes I, III, and IV (*CI*, *CIII*, and *CIV*, respectively).

The concentrations of cross-linkers, and the pH, duration, and temperature of reaction were optimized by varying the conditions and by monitoring the effect of cross-linking on the F-ATPase by SDS-PAGE and blue native-PAGE. The effect of varying the duration and pH of the reaction of DSS(*d*_0_/*d*_12_) with the enzyme is illustrated in Fig. 2 (*lanes a–c* and *d–f*, respectively). The effects of reaction of the enzyme with BS^3^(*d*_0_/*d*_12_), DSG(*d*_0_/*d*_6_), and DSSG(*d*_0_/*d*_6_) for 2 h at pH 8.0 and 37 °C are shown in *lanes g–i* in Fig. 2. The formation of intersubunit cross-links was indicated by the disappearance from the SDS-PAGE gels of the monomeric subunits of the enzyme accompanied by the formation of high molecular weight bands. The optimal conditions were considered to be those where the extent of formation of cross-linked products detected by SDS-PAGE analysis was greatest and the formation of multimeric cross-linked F-ATPase complexes, as detected by blue native-PAGE analysis, was minimal (Fig. 2, *lane j*). Those conditions are 2 μm F-ATPase (1.16 mg/ml) with 2 mm DSS (*d*_0_/*d*_12_) at pH 8.0 for 2 h at 37 °C. In *lanes a–c*, *e*, *f*, and *h* in Fig. 2, it is evident that the a- and c-subunits were unreactive with DSS(*d*_0_/*d*_12_), and in *lane j*, that cross-linking of the enzyme reduced its apparent molecular mass on blue native PAGE gels relative to unreacted enzyme (Fig. 2, *lane k*), presumably because the cross-links make the enzyme more compact.

##### Characterization of Cross-linked Peptides

The number of cross-links identified in each experiment depended upon how the tryptic peptides were generated and fractionated. Fewer cross-linked pairs were identified in samples processed by SDS-PAGE and in-gel digestion than from other samples that were digested with trypsin and analyzed without any further fractionation, probably because of losses of cross-linked peptides in the former procedure during their extraction from gels and subsequent processing. However, there was considerable overlap between the sets of cross-linked peptides that were identified by these means. The most effective procedure was that applied to the tryptic digest of the F-ATPase modified with DSS(*d*_0_/*d*_12_), where cross-linked peptides were separated from unmodified, mono-linked, and loop-linked peptides by cation exchange chromatography at pH 2.7. At this pH value, cross-linked peptide pairs carry four positive charges (or more if histidine residues are present) and so they bind more strongly to a strong cation exchange resin than unmodified, mono-linked, and loop-linked peptides, which carry a minimum of two positive charges. Thus, the cross-linked peptides were recovered in a separate peak eluting after the less positively charged peptides (Fig. 1). Of the 53 cross-linked peptides identified in this peak, (Tables 1 and 2), 30 contained intrasubunit cross-links, and 23 had intersubunit cross-links. In other experiments where the ion exchange step was omitted, 28 peptides cross-linked with DSS were recovered, and 24, 14, and 17 peptides cross-linked with BS^3^, DSG, and DSSG were recovered, respectively. There was no significant difference between the number of cross-linked peptides recovered from the membrane subunits of the F-ATPase reacted with the membrane-permeable reagents, DSS and DSG, and with the membrane-impermeable reagents, BS^3^ and DSSG.

**TABLE 1:** **Cross-linked tryptic peptides in the membrane extrinsic region of the bovine F-ATPase** The cross-links were produced by reaction of the enzyme with DSS(*d*_0_/*d*_12_), and the peptides were fractionated by ion exchange chromatography.

Subunits	Cross-linked sequences	Lys-Lys*^a^*	Cα-Cα*^b^*
α-α	VVDALGNAIDG**K**GPIGSK-STVAQLV**K**R	118–218*^c,d^*	14.9
α-α	GPIGS**K**AR-DNG**K**HALIIYDDLSK	124–262*^c,e^*	17.6
α-α	VGL**K**APGIIPRISVR-RFNDGTDE**K**K	132–196	25.4
α-α	FNDGTDE**K**K-DNG**K**HALIIYDDLSK	196–262*^c,d^*	7.4
α-α	FNDGTDE**K**K-TDG**K**ISEESDAK	196–488	19.3
α-α	AM**K**QVAGTMK-TDG**K**ISEESDAK	384–488*^c,d,e^*	8.8
α-α	GYLD**K**LEPSK-L**K**EIVTNFLAGFEA	455–498*^c,d,e^*	15.1
α-α	FENAFLSHVISQHQGALLS**K**IR-TDG**K**ISEESDAK	482–488*^d,e^*	8.4
α-β	VVDALGNAIDG**K**GPIGSK-ITTT**K**K	118–304*^c,d,e^*	16.8
α-β	RSTVAQLV**K**R-ITTT**K**K	218–304*^c,d,e^*	15.0
α-γ	VGSAAQTRAM**K**QVAGTMK-ATL**K**DITR	384–4	39.1
α-oscp	LI**K**EGDIVKR-YVDMSA**K**TK	83–176	21.0
α-b	VVDALGNAIDG**K**GPIGSK-EV**K**NR	118–152	21.6
α-d	GYLD**K**LEPSK-NQ**K**AVANSLK	455–24	32.3
β-β	AAQASPSP**K**AGATTGR-GQ**K**VLDSGAPIR	9–78*^d^*	–*^f^*
β-β	VVDLLAPYA**K**GGK-LVPL**K**ETIK	152–439	18.2
β-β	FLSQPFQVAEVFTGHLG**K**LVPLK-AD**K**LAEEHS	430–476*^d^*	18.5
β-δ	**K**IQR-ANLE**K**AQSELLGAADEATR	413–114	57.3
γ-γ	ATL**K**DITR-SI**K**NIQK	4–14	15.3
γ-γ	SI**K**NIQK-IT**K**SMK	14–21*^d^*	10.8
γ-γ	ADI**K**TPEDK-SEAANLAAAG**K**EVK	58–101	14.3
γ-ϵ	MVAAA**K**YAR-ANAM**K**TSGSTIK	30–36*^c,d,e^*	14.4
γ-ϵ	EL**K**PAR-ANAM**K**TSGSTIK	39–36	16.3
γ-ϵ	THSDQFLVTF**K**EVGR-IV**K**VK	129–46*^e^*	10.5
δ-ϵ	ANLE**K**AQSELLGAADEATR-YSQICA**K**AVR	114–20*^c,e^*	12.7
δ-ϵ	ANLE**K**AQSELLGAADEATR-TEF**K**ANAMK	114–31*^e^*	21.0
δ-ϵ	ANLE**K**AQSELLGAADEATR-DAL**K**TEFKANAMK	144–27	18.3
ϵ-ϵ	YSQICA**K**AVR-TEF**K**ANAMK	20–31*^c,d^*	13.0
ϵ-ϵ	YSQICA**K**AVR-ANAMKTSGSTIK	20–36*^c^*	13.8
oscp-oscp	VGQIL**K**EPK-SV**K**VK	47–65	15.5
oscp-oscp	EP**K**MAASLLNPYVK-V**K**SLSDMTAK	50–67	16.5
oscp-oscp	EP**K**MAASLLNPYVK-SLSDMTA**K**EK	50–75*^c^*	16.3
oscp-oscp	MAASLLNPYV**K**R-V**K**SLSDMTAK	61–67*^c^*	11.0
b-b	VVQSISAQQE**K**ETIAK-CIADL**K**LLSK	191–202*^d^*	16.3
b-d	QIQDAIDME**K**SQQALVQK-SCAEFLTQS**K**TR	112–108*^d^*	11.8
b-F_6_	VVQSISAQQE**K**ETIAK-N**K**ELDPVQK	191–2	–*^g^*
b-F_6_	VVQSISAQQE**K**ETIAK-LFVD**K**IR	191–14	14.7
d-d	**K**LALK-LATLPE**K**PPAIDWAYYK	4–47*^c,e^*	12.9
d-d	**K**LALK-FNAL**K**VPIPEDK	4–77	18.3
d-d	LATLPE**K**PPAIDWAYYK-FNAL**K**VPIPEDK	47–77	13.0
d-d	ANVA**K**AGLVDDFEK-**K**FNALK	62–72*^d^*	16.0
d-d	AGLVDDFE**K**K-FNAL**K**VPIPEDK	71–77	12.0
d-d	SCAEFLTQS**K**TR-IQEYE**K**ELEK	108–116	12.2
d-F_6_	ANVA**K**AGLVDDFEK-L**K**QMYGK	62–47	17.8

*^a^* Sequence numbers of cross-linked lysines. The hyphens separate the two peptides, and cross-linked residues are bold.

*^b^* Measured in Å in the structure of the bovine F_1_-c_8_-peripheral stalk complex.

*^c^* Cross-link also found in bovine F-ATPase modified with DSG(*d*_0_*d*_6_).

*^d^* Cross-link also found in bovine F-ATPase modified with BS^3^(*d*_0_*d*_12_).

*^e^* Cross-link also found in bovine F-ATPase modified with DSSG(*d*_0_*d*_6_).

*^f^* The N-terminal region of β-subunits is disordered.

*^g^* Residue 2 of subunit F_6_ is not resolved.

**TABLE 2:** **Mass spectrometric characterization of cross-linked tryptic peptides in the membrane extrinsic region of the bovine F-ATPase** The cross-links were produced by reaction of the enzyme with DSS(*d*_0_/*d*_12_), and the peptides were fractionated by ion exchange chromatography.

Subunits	Lys-Lys*^a^*	Score	*m*/*z*	MH^+^	Charge	ppm
				*Da*		
α-α	118–218*^b,c^*	758	950.54217	2849.61197	3	0.89
α-α	124–262*^b,d^*	738	875.47418	2624.40800	3	1.45
α-α	132–196	450	974.88971	2922.65459	3	0.78
α-α	196–262*^b,c^*	691	964.81470	2892.42956	3	1.19
α-α	196–488	627	824.05456	2470.14914	3	0.98
α-α	384–488*^b,c,d^*	839	827.74683	2481.22595	3	1.95
α-α	455–498*^b,c,d^*	486	946.84473	2838.51965	3	0.76
α-α	482–488*^c,d^*	635	964.50415	3854.99479	4	0.65
α-β	118–304*^b,c,d^*	852	847.15118	2539.43900	3	1.93
α-β	218–304*^b,c,d^*	436	497.30722	1986.20707	4	0.04
α-γ	384–4	615	723.14758	2889.56851	4	1.26
α-oscp	83–176	573	784.10754	2350.30808	3	1.30
α-b	118–152	725	831.79480	2493.36986	3	1.11
α-d	455–24	745	787.10058	2359.28720	3	0.20
β-β	9–78*^c^*	478	712.88782	2848.52947	4	0.69
β-β	152–439	496	836.84064	2508.50738	3	0.92
β-β	430–476*^c^*	707	923.74963	3691.976671	4	0.91
β-δ	413–114	621	890.14807	2668.42967	3	1.22
γ-γ	4–14	782	629.04211	1885.11179	3	0.05
γ-γ	14–21*^c^*	480	558.99921	1674.98309	3	0.49
γ-γ	58–101	682	838.11029	2512.31633	3	0.91
γ-ϵ	30–36*^b,c,d^*	572	776.08063	2326.22735	3	0.69
γ-ϵ	39–36	512	692.37775	2075.11871	3	1.07
γ-ϵ	129–46*^d^*	595	829.80475	2487.39971	3	1.11
δ-ϵ	114–20*^b,d^*	645	1107.23755	3319.69811	3	0.92
δ-ϵ	114–31*^d^*	575	1055.20410	3163.59776	3	1.21
δ-ϵ	144–27	579	902.46393	3606.83391	4	0.55
ϵ-ϵ	20–31*^c,d^*	899	791.40918	2372.21300	3	1.22
ϵ-ϵ	20–36*^d^*	711	847.77789	2541.31913	3	1.09
oscp-oscp	47–65	768	570.35724	1709.05718	3	0.04
oscp-oscp	50–67	949	926.50006	2777.48564	3	1.05
oscp-oscp	50–75*^d^*	1083	936.49115	2807.45891	3	0.71
oscp-oscp	61–67*^d^*	577	860.47052	2579.39702	3	1.35
b-b	191–202*^c^*	817	1019.57013	3056.69585	3	1.54
b-d	112–108*^c^*	1211	910.21136	3637.82363	4	0.99
b-F_6_	191–2	816	989.54022	2966.60612	3	0.57
b-F_6_	191–14	669	929.52832	2786.57042	3	1.52
d-d	4–47*^b,d^*	620	672.13818	2685.53091	4	1.62
d-d	4–77	671	694.08649	2080.24493	3	1.60
d-d	47–77	559	871.72638	3483.88371	4	0.68
d-d	62–72*^c^*	622	778.76239	2334.27263	3	0.98
d-d	71–77	712	877.14307	2629.41467	3	0.90
d-d	108–116	839	958.48065	2873.42741	3	1.29
d-F_6_	62–47	690	827.77423	2481.30815	3	0.97

*^a^* Sequence numbers of cross-linked lysines.

*^b^* Cross-link also found in bovine F-ATPase modified with DSG(*d*_0_*d*_6_).

*^c^* Cross-link also found in bovine F-ATPase modified with BS^3^(*d*_0_*d*_12_).

*^d^* Cross-link also found in bovine F-ATPase modified with DSSG(*d*_0_*d*_6_).

##### Cross-links in the Membrane Extrinsic Region of the F-ATPase

Forty-four cross-linked peptide pairs were identified as originating from membrane-extrinsic subunits of the enzyme. They are summarized in Table 1 together with the Cα distances between the cross-linked lysine residues measured in the structure of the bovine F-ATPase, and the corresponding mass spectrometry data are summarized in Table 2. The lengths of cross-linkers are given in the experimental section. In 38 of them both lysines are resolved in the structure, and the formation of the cross-links is compatible with the inter-Cα distances. For a 39th cross-link between αLys-455 and Lys-24 in subunit d, the inter-Cα distance of 32.3 Å is only slightly above the maximum value of 27.4 Å, and a minor conformational change in the determined structure of the enzyme would allow this cross-link to form. In two other cross-links between the F_1_ domain and the peripheral stalk, one of the reacted lysine residues is in a region that is locally unresolved in the structure, although adjacent structural elements are well defined. One such cross-link bridges between unresolved residue βLys-9 in the N-terminal region of the subunit and residue βLys-78, which is resolved in the six-stranded β-barrel that forms part of the “crown” of the F_1_ domain. The Cα distances between the resolved residue βThr-13, which is close to βLys-9 in the sequence of the subunit, and βLys-78 are 10 Å, 10 Å, and 10.2 Å in the β_DP_-, β_TP_-, and β_E_-subunits, respectively. Therefore, the formation of a cross-link bridging between βLys-9 and βLys-78 is compatible with the structure. The second cross-link involves the unresolved residue Lys-2 in subunit F_6_ and the resolved residue Lys-191 in subunit b. It is clear from the structure of the enzyme that residue Lys-2 in subunit F_6_ and residue Lys-191 in the C-terminal region of subunit b are in close proximity (the estimated distance is ∼25 Å), and the formation of this cross-link is consistent with this conclusion.

Three other cross-links are incompatible with the structure of the monomeric bovine F-ATPase, and each was observed once only (see Table 1). One of them bridges between βLys-413 and δLys-114, whereas the Cα distance between the two lysine residues in the structure is 57.3 Å. This cross-link is likely to have formed between two different F-ATPase complexes, and the presence of a small amount of the oligomeric complex in the native gel of the modified complex (Fig. 2, *lane j*) is consistent with this suggestion. Another incompatible cross-link bridges between αLys-132 and αLys-196, two surface residues in the nucleotide binding domain of the subunit. Although the inter-Cα value given in Table 1 is apparently within the range of acceptable distances, this distance has been measured along the direct path between the two Cα-atoms, which is impeded by a loop involving residues 309–321 of the α-subunit, and the unimpeded Cα distance is considerably longer. The third structurally incompatible cross-link is between αLys-384 and γLys-4. The former residue is exposed at the entrances to catalytic interfaces between the α-subunits and the adjacent β-subunit, whereas the latter residue is in the N-terminal α-helix of the γ-subunit. This α-helix is one of the two α-helical elements in the central shaft of the rotor found in the aqueous cavity in the core of the F_1_ domain. In one of the three catalytic α-β interfaces, between α_DP_ and β_DP_, there is a direct unimpeded line of sight between the two residues, but the gap narrows to ∼6 Å, and the inter-Cα distance is 38.4 Å. These last two structurally incompatible cross-links could only have formed by reaction with disrupted F-ATPase complexes. Despite these three aberrant cross-links, the overwhelming majority, 93%, of the observed linkages were compatible with the atomic structure of the F-ATPase. Their main value is that they provide reassurance that any cross-links observed in regions of the F-ATPase that are not represented in the current structural model are likely to have validity and that they represent contributions toward defining the organization of subunits in the unresolved region.

##### Cross-links in the Membrane Domain of the F-ATPase

Eight intersubunit and four intrasubunit cross-links were identified among the tryptic peptides from the F-ATPase that had been cross-linked with the four reagents. The majority of them were identified in the digest of the DSS cross-linked enzyme that was fractionated by ion exchange chromatography. They are summarized in Table 3, and the corresponding mass spectrometry data are summarized in Table 4.

**TABLE 3:** **Cross-links to tryptic peptides in the subunits of the membrane intrinsic region of the bovine F-ATPase**

Subunits	Cross-linked sequences	Lys-Lys*^a^*
e-e	LAAEE**K**K-DEQ**K**R	47–54*^b,c,d,e^*
e-e	LAAEEK**K**K-RDEQ**K**R	48–54*^b,c,d,e^*
e-f	LAAEE**K**K-**K**YH	47–85*^b,c,d,e^*
f-f	E**K**K-LLEV**K**LGELPSWILMR	9–15*^b,c,d,e^*
f-g	LLEV**K**LGELPSWILMR-TGSF**K**QLTVK	15–65*^e^*
ATP8-b	ML**K**QNTPWETK-SQQALVQ**K**R	46–120*^b^*
ATP8-d	ML**K**QNTPWETK-NQ**K**AVANSLK	46–24*^b^*
ATP8-F_6_	QNTPWET**K**WTK-FEVVE**K**PQS	54–73*^b,e^*
6.8PL-6.8PL	SAD**K**R-AL**K**ASSAAPAHGHH	43–49*^b,e^*
6.8PL-e	AL**K**ASSAAPAHGHH-DEQ**K**R	49–54*^b^*
6.8PL-f	AL**K**ASSAAPAHGHH-EL**K**HER	49–79*^b^*
6.8PL-DAPIT	AL**K**ASSAAPAHGHH-TPAV**K**AT	49–55*^b^*

*^a^* Sequence numbers of cross-linked lysines. The hyphens separate the two peptides, and the cross-linked residues are bold.

*^b^* Cross-links identified in F-ATPase reacted with DSS(*d*_0_/*d*_12_).

*^c^* Cross-links identified in F-ATPase reacted with DSG(*d*_0_*d*_6_).

*^d^* Cross-links identified in F-ATPase reacted with DSSG(*d*_0_*d*_6_).

*^e^* Cross-links identified in F-ATPase reacted with BS^3^(*d*_0_*d*_12_).

**TABLE 4:** **Mass spectrometric characterization of cross-linked tryptic peptides in the subunits of the membrane intrinsic region of the bovine F-ATPase**

Subunits	Lys-Lys*^a^*	Score	*m*/*z*	MH^+^	Charge	ppm
				*Da*		
e-e	47–54*^b,c,d,e^*	927	800.93109	1600.85491	2	0.53
e-e	48–54*^b,c,d,e^*	775	629.02154	1885.05008	3	0.03
e-f	47–85*^b,c,d,e^*	765	686.87793	1372.74859	2	1.10
f-f	9–15*^b,c,d,e^*	1013	813.47479	2438.40983	3	0.30
f-g	15–65*^e^*	687	1048.27112	3142.79880	3	0.57
ATP8-b	46–120*^b^*	548	862.79572	2586.37259	3	0.68
ATP8-d	46–24*^b^*	868	862.46423	2585.37815	3	0.99
ATP8-F_6_	54–73*^b,e^*	633	873.44446	2618.31882	3	1.97
6.8PL-6.8PL	43–49*^b,e^*	693	690.02801	2068.06949	3	0.61
6.8PL-e	49–54*^b^*	504	542.53105	2167.09878	4	0.69
6.8PL-f	49–79*^b^*	422	576.55573	2303.20107	4	0.33
6.8PL-DAPIT	49–55*^b^*	560	727.05920	2179.16306	3	0.58

*^a^* Sequence numbers of cross-linked lysines.

*^b^* Cross-links identified in F-ATPase reacted with DSS(*d*_0_*d*_12_).

*^c^* Cross-links identified in F-ATPase reacted with DSG(*d*_0_*d*_6_).

*^d^* Cross-links identified in F-ATPase reacted with DSSG(*d*_0_*d*_6_).

*^e^* Cross-links identified in F-ATPase reacted with BS^3^(*d*_0_*d*_12_).

**FIGURE 3.**
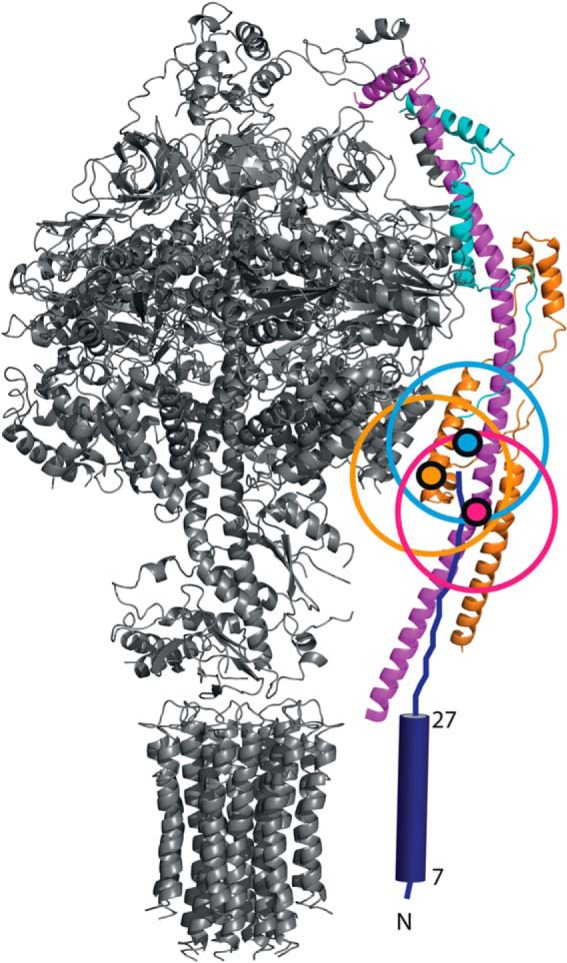
**Intersubunit cross-links between the C-terminal region of mitochondria-encoded subunit ATP8 and nuclear encoded subunits b, d, and F_6_ in the structure of the bovine F-ATPase.** The structures of the membrane extrinsic F_1_ domain and the attached oscp *(top*) and the c_8_-ring in the membrane domain of the enzyme (*bottom*) are shown in *black*. In the peripheral stalk of the complex (*right*), the membrane extrinsic region of subunit b is *magenta*, subunit F_6_ is *blue*, and subunit d is *orange*. Subunit ATP8 is deep *blue*, and its predicted transmembrane α-helix from residues 8–29 is depicted as a *cylinder*. Except for the c-ring, the region occupied by membrane components of the enzyme is not shown. The secondary structure of residues 29–66 of subunit ATP8 is not known, and this region is shown in an extended form reaching up so as to place lysine residues 46 and 54 in the vicinity of the residues to which they are cross-linked, residues 120, 24, and 73 (indicated by the *magenta*, *orange*, and *blue dots* in subunits b, d, and F_6_, respectively). The *colored circles around each dot* have a diameter of 20 Å, and they represent the region in which each cross-linked residue in ATP8 is likely to be found in the structure.

Three of the intersubunit cross-links were between subunit ATP8 and components of the peripheral stalk domain. One of them links residue Lys-54 of subunit ATP8 to residue Lys-73 of the F_6_ subunit. The two others involve links from residue Lys-46 of ATP8, one to residue Lys-120 of subunit b and the other to residue Lys-24 of subunit d (Fig. 3).

**FIGURE 4.**
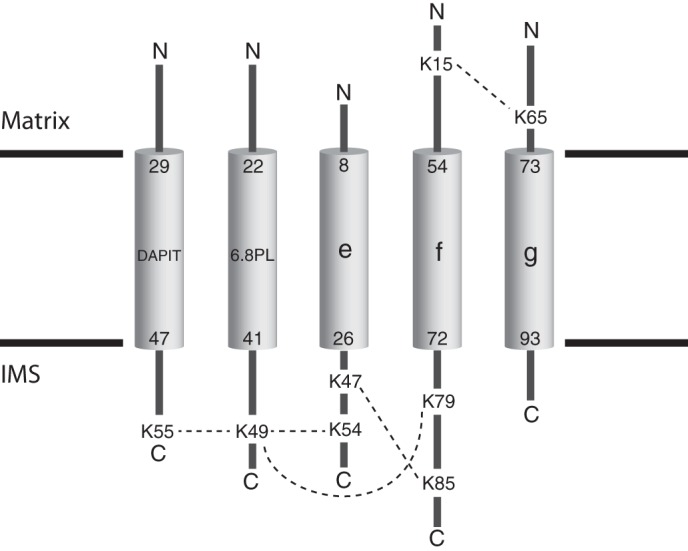
**Intersubunit cross-links detected in the nuclear encoded supernumerary subunits of the bovine F-ATPase.** The *cylinders* represent the transmembrane α-helices predicted to be present in each of subunits e, f, g, DAPIT, and 6.8PL. The positions of the α-helices in the sequences of the subunits are indicated by the *numbers at the top and bottom of each cylinder*. The *dashed lines* represent the intersubunit cross-links, introduced by reaction of the bifunctional cross-linking agents with the numbered lysine residues in the N- and C-terminal regions of each subunit. *IMS*, intermembrane space.

Five of the intersubunit cross-links involved lysine residues in the five supernumerary subunits e, f, g, DAPIT, and 6.8PL (see Fig. 4). All fall in regions of the proteins that are predicted to be membrane extrinsic. Three involve links between residue Lys-49 in the C-terminal region of the 6.8PL subunit with lysine residues in the C-terminal regions of the DAPIT, e, and f subunits. Other cross-links bridge between the C-terminal regions of subunits e and f and the N-terminal regions of subunits f and g.

## Discussion

### 

#### 

##### Involvement of Subunit ATP8 in the Bovine Peripheral Stalk

**FIGURE 5.**
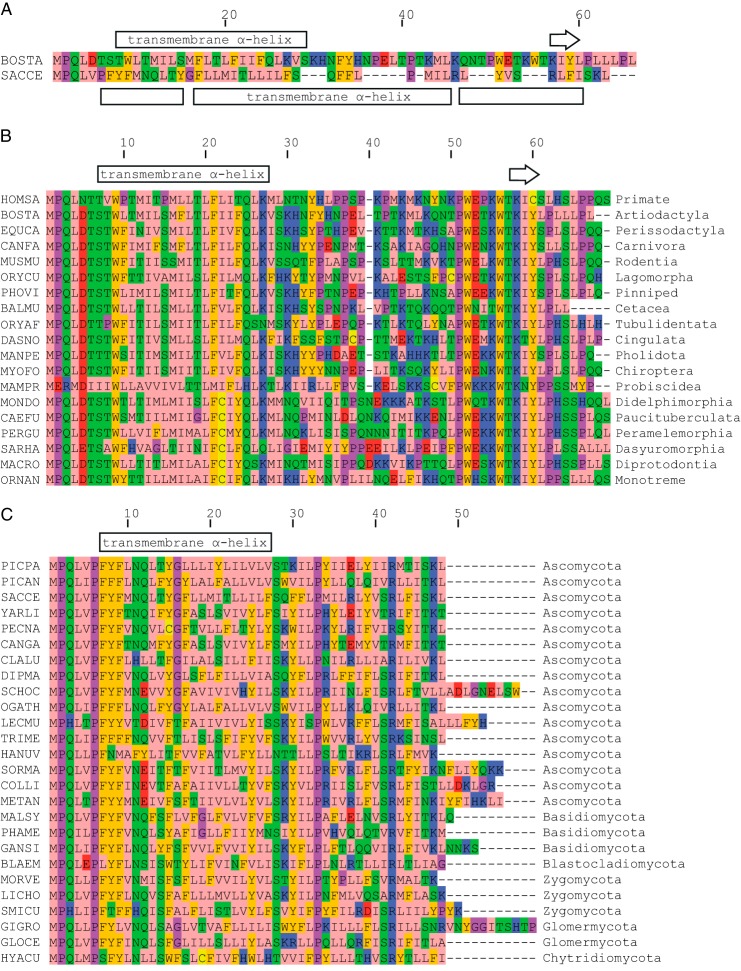
**Conservation of sequences of ATP8 subunits.** Residues are colored as follows: *pink*, hydrophobic; *orange*, aromatic; *blue*, basic; *red*, acidic; *green*, hydrophilic; *magenta*, proline and glycine. *Yellow* denotes cysteine residues. *A*, comparison of the sequences of the bovine and yeast ATP8 subunits. The predicted secondary structure of the bovine protein is shown *above the sequences*. The *arrow* represents a short predicted β-strand. The regions before, between, and after the α-helices and β-sheet are predicted to be extended. *BOSTA*, *Bos taurus*; *SACCE*, *S. cerevisiae. B* and *C*, conservation of sequences of selected mammalian and fungal ATP8 subunits. In *B* and *C*, the orders represented by the species are shown on the *right. B*: *HOMSA*, *Homo sapiens*; BOSTA, *B. taurus*; *EQUCA*, *Equus caballus* (horse); *CANFA*, *Canis lupus familiaris* (dog); *MUSMU*, *Mus musculus* (mouse); *ORYCU*, *Oryctolagus cuniculus* (rabbit); *PHOVI*, *Phoca vitulina* (harbor seal); *BALMU*, *Balaenoptera musculus* (blue whale); *ORYAF*, *Orycteropus afer* (aardvark); *DANO*, *Dasypus novemcinctus* (nine-banded armadillo); *MANPE*, *Manis pentadactyla* (Chinese pangolin); *MYOFO*, *Myotis formosus* (Hodgson's bat); *MAMPR*, *Mammuthus primigenius* (woolly mammoth); *MONDO*, *Monodelphis domestica* (gray short tailed opossum); *CAEFU*, *Caenolestes fuliginosus* (silky shrew opossum); *PERGU*, *Perameles gunnii* (eastern barred bandicoot); *SARHA*, *Sarcophilus harrisii* (Tasmanian devil); *MACRO*, *Macropus robustus* (common wallaroo); *ORNAN*, *Ornithorhynchus anatinus* (duck-billed platypus). *C*: *PICPA*, *Pichia pastoris*; *PICAN*, *Pichia angusta*; *SACCE*, *S. cerevisiae*; *YARLI*, *Yarrowia lipolytica*; *PECNA*, *Pneumocystis carinii*; *CANGA*, *Candida galli*; *CLALU*, *Clavispora lusitaniae*; *DIPMA*, *Dipodascus magnusii*; *SCHOC*, *Schizosaccharomyces octosporus*; *OGATH*, *Ogataea thermophila*; LECMU, *Lecanicillium muscarium*; *TRIME*, *Trichophyton mentagrophytes*; *HANUV*, *Hanseniaspora uvarum*; *SORMA*, *Sordaria macrospora*; *COLLI*, *Colletotrichum lindemuthianum*; *METAN*, *Metarhizium anisopliae*; *MALSY*, *Malassezia sympodialis*; *PHAME*, *Phakopsora meibomiae*; *GANSI*, *Ganoderma sinense*; *BLAEM*, *Blastocladiella emersonii*; *MORVE*, *Mortierella verticillata*; *LICHO*, *Lichtheimia hongkongensis*; *SMICU*, *Smittium culisetae*; *GIGRO*, *Gigaspora rosea*; *GLOCE*, *Glomus cerebriforme*; *HYACU*, *Hyaloraphidium curvatum.*

Since its discovery as a subunit of the bovine and yeast F-ATPases (12, 33, 34), subunit ATP8 (also known as A6L in mammals and Aap1 in *Saccharomyces cerevisiae*) has remained a rather mysterious component of the enzyme complex. It is not found in eubacterial and chloroplast enzymes, and therefore, it was classified as a supernumerary subunit, apparently not required for the core ATP synthetic and hydrolytic functions of F-ATPases. The subunit is encoded in the mitochondrial DNA of many, but not all, eukaryotic species (35), and in mammals, the genes for ATP8 and ATPase-6 (or subunit a) overlap (12, 36). The bovine protein is probably folded into a single transmembrane α-helix from residues 8–29 followed by a hydrophilic extension up to its C terminus at residue 66 (Fig. 5*A*). This region from residue 30 to 66 is predicted to have an extended conformation, except for residues 57–61, which may form a short β-sheet. The region consisting of residues 51–63 is well conserved in mammals (Fig. 5*B*). In contrast, subunit ATP8 in *S. cerevisiae* has a C-terminal extension that is predicted to be mostly α-helical. It is significantly shorter and poorly related to the same region of the mammalian proteins, although the sequences of ATP8 proteins are well conserved among the fungi (Fig. 5*C*). On the basis of cysteine scanning mutagenesis and reaction with fluorescein-5-maleimide, residues 1–14 of yeast ATP8 have been proposed to be exposed to the intermembrane space followed by a transmembrane α-helix from residues 15–35 (37). A short β-strand is predicted around residue Arg-42, and this residue is conserved throughout fungi (Fig. 5*C*). Despite these differences, proteolytic digestion studies conducted on bovine mitochondrial membranes (38, 39) and cross-linking studies on the yeast enzyme (40) have shown that the bovine and yeast proteins have a common topography with their N-terminal regions in the intermembrane space and their C-terminal regions on the matrix side of the inner mitochondrial membrane.

In the current work new information about the location of bovine subunit ATP8 in the F-ATPase has come from an extensive study of covalent cross-linking conducted on the intact purified enzyme with bi-functional agents. Among the many identified cross-links, residues Lys-46 and Lys-54 of ATP8 were found to be linked to three lysine residues, one in each of subunits b, d, and F_6_ in the peripheral stalk of the enzyme. These cross-links confirm that the C-terminal region of bovine ATP8 is exposed in the matrix of the organelle, and they demonstrate that this region of ATP8 is in the vicinity of the peripheral stalk. The shorter and poorly related C-terminal region of yeast subunit ATP8 has been proposed to interact with subunits b and d in the yeast enzyme (37). Two of the cross-links in the bovine enzyme involve the structurally defined residues Lys-120 and Lys-24 of subunits b and d, respectively, both of which are found ∼60 Å from the membrane domain of the enzyme. Therefore, residues Lys-46 and Lys-54 of ATP8 are nearby (see Fig. 3), and the C-terminal region of bovine subunit ATP8 extends ∼60–70 Å upwards from the membrane domain, probably along the axis of the peripheral stalk defined by the long α-helix in subunit b. The third cross-link is between residue Lys-54 in ATP8 and residue Lys-73 in subunit F_6_ (Fig. 3). Bovine subunit F_6_ is 76 amino acids long, and in the peripheral stalk, it is folded into two α-helices from residues 4–25 and 33–51 linked by an extended loop region from residues 26–32. Residue 51 is followed by an extended region, which is resolved up to residue Glu-69. The cross-linked residue Lys-73 of F_6_ is nearby and is estimated to be ∼70 Å from the surface of the membrane domain of the enzyme, in accordance with the position deduced from the structurally defined lysine residues in subunits b and d. The only known human pathogenic mutation specific to subunit ATP8 is associated with infantile cardiomyopathy, and it leads to the truncation of the subunit at residue 54 and impairment of ATP synthesis (41). Therefore, in mammalian mitochondrial enzymes subunit ATP8 appears to have a fundamental role in either the synthesis of ATP by mitochondrial F-ATPases or the assembly of the complex or both. If the C-terminal region of subunit ATP8 is, as proposed, an intrinsic component of the peripheral stalk, truncation of the subunit would disrupt the integrity of the peripheral stalk and impair ATP synthesis by uncoupling the catalytic sites in the F_1_ domain from the proton motive force, generated across the inner mitochondrial membrane by respiration. Similar proposals have been made that the yeast ATP8 subunit is a component of the peripheral stalk of the yeast F-ATPase (37, 40) and that it is required for the assembly of the complex (42, 43). Thus, despite significant differences in the lengths and sequences of mammalian and yeast ATP8 subunits, it is likely that they have related roles in the enzyme complexes.

##### Organization of Supernumerary Subunits

**FIGURE 6.**
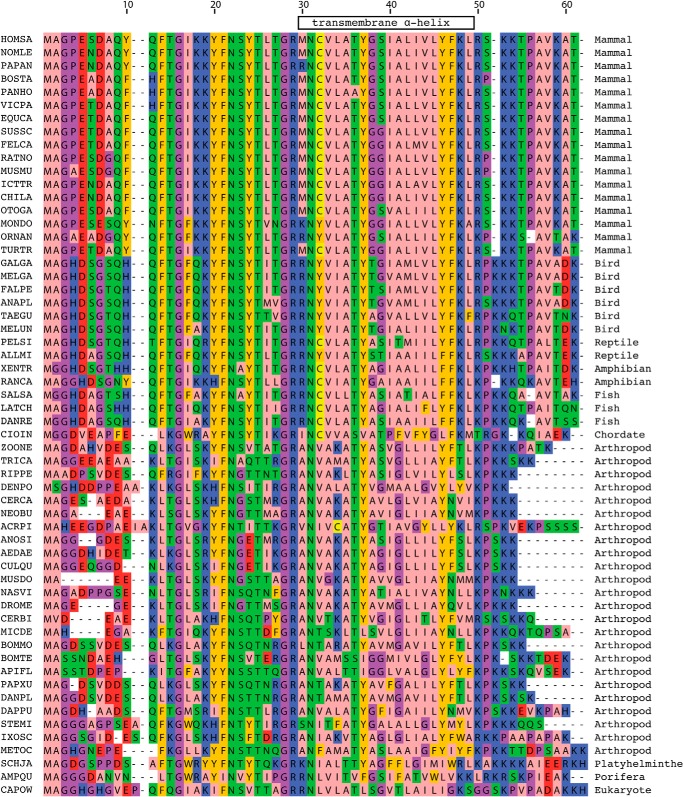
**Conservation of the sequences of the DAPIT subunit in metazoan F-ATPases.** For details of the color scheme see the legend to Fig. 5. *HOMSA*, *H. sapiens* (human); *BOSTA*, *B. taurus* (cow); *SUSSC*, *Sus scrofa* (wild boar); *NOMLE*, *Nomascus leucogenys* (Northern white-cheeked gibbon); *PAPAN*, *Papio anubis* (olive baboon); *PANHO*, *Pantholops hodgsonii* (chiru); *VICPA*, *Vicugna pacos* (alpaca); *EQUCA*, *E. caballus* (horse); *FELCA*, *Felis catus* (cat); *RATNO*, *Rattus norvegicus* (rat); *MUSMU*, *M. musculus* (mouse); *ICTTR*, *Ictidomys tridecemlineatus* (squirrel); *CHILA*, *Chinchilla lanigera* (long-tailed chinchilla); *OTOGA*, *Otolemur garnettii* (Garnett's greater bushbaby); *MONDO*, *M. domestica* (gray short-tailed opossum); *ORNAN*, *O. anatinus* (duckbill platypus); *TURTR*, *Tursiops truncatus* (Atlantic bottle-nosed dolphin); *GALGA*, *Gallus gallus* (chicken); *MELGA, Meleagris gallopavo* (common turkey); *FALPE*, *Falco peregrinus* (peregrine falcon); ANAPL, *Anas platyrhynchos* (domestic duck); *TAEGU*, *Taeniopygia guttata* (zebra finch); *MELUN*, *Melopsittacus undulatus* (budgerigar); *PELSI*, *Pelodiscus sinensis* (Chinese softshell turtle); *ALLMI*, *Alligator mississippiensis* (American alligator); *XENTR*, *Xenopus tropicalis* (clawed frog); *RANCA*, *Rana catesbeiana* (American bullfrog); *SALSA*, *Salmo salar* (salmon); *LATCH*, *Latimeria chalumnae* (West Indian ocean coelacanth); *DANRE*, *Danio rerio* (zebrafish); *CIOIN*, *Ciona intestinalis* (transparent sea squirt); *ZOONE*, *Zootermopsis nevadensis* (termite); *TRICA*, *Tribolium castaneum* (red flour beetle); *RIPPE*, *Riptortus pedestris* (bean bug); *DENPO*, *Dendroctonus ponderosae* (mountain pine beetle); *CERCA*, *Ceratitis capitata* (medfly); *NEOBU*, *Neobellieria bullata* (flesh fly); *ACRPI*, *Acyrthosiphon pisum* (pea aphid); *ANOSI*, *Anopheles sinensis* (mosquito); *AEDAE*, *Aedes aegypti* (yellow fever mosquito); *CULQU*, *Culex quinquefasciatus* (house mosquito); *MUSDO*, *Musca domestica* (housefly); *NASVI*, *Nasonia vitripennis* (jewel wasp); *DROME*, *Drosophila melanogaster* (fruit fly); *CERBI*, *Cerapachys biroi* (clonal raider ant); *MICDE*, *Microplitis demolitor* (parasitic wasp); *BOMMO*, *Bombyx mori* (silkmoth); *BOMTE*, *Bombus terrestris* (bumblebee); APIFL, *Apis florea* (dwarf honey bee); *PAPXU*, *Papilio xuthus* (Asian swallowtail); DANPL, *Danaus plexippus* (monarch butterfly); *DAPPU*, *Daphnia pulex* (water flea); *STEMI*, *Stegodyphus mimosarum* (social spider); *IXOSC*, *Ixodes scapularis* (tick); *METOC*, *Metaseiulus occidentalis* (mite); *SCHJA*, *Schistosoma japonicum* (blood fluke); *AMPQU*, *Amphimedon queenslandica* (sea sponge); *CAPOW*, *Capsaspora owczarzaki* (filasterean amoeba).

**FIGURE 7.**
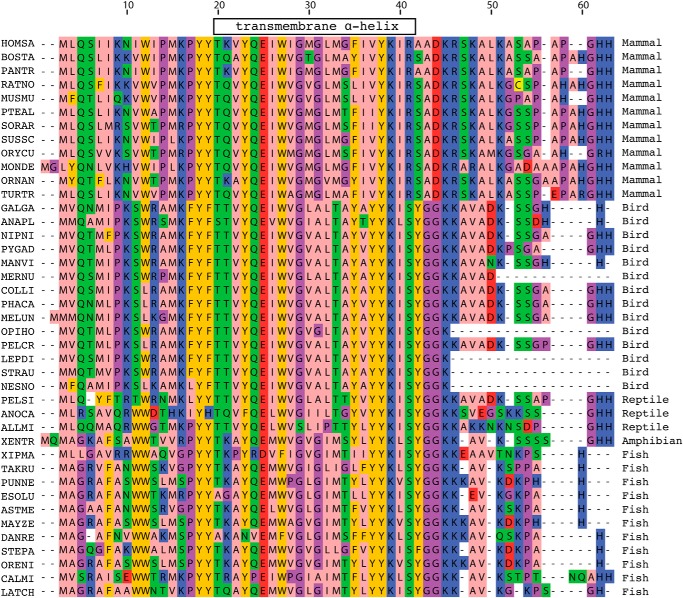
**Conservation of the sequences of the 6.8PL subunit in vertebrate F-ATPases.** For details of the color scheme, see the legend to Fig. 5. *HOMSA*, *H. sapiens* (human); *BOSTA*, *B. taurus* (cow); *PANTR*, *Pan troglodytes* (chimpanzee); *RATNO*, *R. norvegicus* (rat); *MUSMU*, *M. musculus* (mouse); *PTEAL*, *Pteropus alecto* (black flying fox); *SORAR*, *Sorex araneus* (shrew); *SUSSC*, *S. scrofa* (wild boar); *ORYCU*, *O. cuniculus* (rabbit); *MONDE*, *M. domestica* (gray short-tailed opossum); *ORNAN*, *O. anatinus* (duckbill platypus); *TURTR*, *T. truncatus* (bottle nose dolphin); *GALGA*, *G. gallus* (chicken); *ANAPL*, *A. platyrhynchos* (domestic duck); *NIPNI*, *Nipponia nippon* (crested ibis); *PYGAD*, *Pygoscelis adeliae* (Adelie penguin); *MANVI*, *Manacus vitellinus* (golden-collared manakin); *MERNU*, *Merops nubicus* (northern carmine bee eater); *COLLI*, *Columba livia* (pigeon); *PHACA*, *Phalacrocorax carbo* (cormorant); *MELUN, M. undulatus* (budgerigar); *OPIHO*, *Opisthocomus hoazin* (hoatzin); *PELCR*, *Pelecanus crispus* (pelican); *LEPDI, Leptosomus discolor* (cuckoo); *STRAU*, *Struthio camelus australis* (ostrich); *NESNO*, *Nestor notabilis* (kea); *PELSI*, *P. sinensis* (Chinese softshell turtle); *ANOCA*, *Anolis carolinensis* (green anole); *ALLMI*, *A. mississippiensis* (American alligator); *XENTR*, *X. tropicalis* (clawed frog); *XIPMA*, *Xiphophorus maculatus* (platy); *TAKRU*, *Takifugu rubripes* (pufferfish); *PUNNE*, *Pundamilia nyererei* (flame black cichlid); *ESOLU*, *Esox lucius* (Northern pike); ASTME, *Astyanax mexicanus* (Mexican tetra); *MAYZE*, *Maylandia zebra* (Zebra mbuna); *DANRE*, *D. rerio* (zebrafish); *STEPA*, *Stegastes partitus* (bicolor damselfish); *ORENI*, *Oreochromis niloticus* (Nile tilapia); *CALMI*, *Callorhinchus milii* (Australian ghost shark); *LATCH*, *L. chalumnae* (West Indian ocean coelacanth).

The presence of the supernumerary subunits e, f, and g in the membrane domains of F-ATPases was demonstrated first in the bovine enzyme (2, 13) and subsequently in the enzyme from *S. cerevisiae* (15, 44). All three subunits are predicted to contain a single transmembrane α-helix (see Fig. 4), and the sequences of the bovine and yeast e, f, and g subunits are conserved or conservatively substituted in 26%, 34%, and 46% of their residues, respectively. The predicted secondary structures of the bovine and yeast orthologs are similar but not entirely concordant. Since their discovery, the roles of the yeast subunits have been studied much more extensively than their bovine orthologs. Yeast subunits e and g are involved in the formation of dimers of the enzyme in the mitochondrial cristae (15, 45, 46), and they are oriented with their N-terminal regions in the mitochondrial matrix and their C-terminal regions in the intermembrane space of the organelle (37, 47). A G*XXX*G sequence motif in residues 14–18 of the yeast e-subunit forms a homodimeric α-helical coiled-coil involved in holding two F-ATPase complexes together (45). However, the motif is not conserved in the bovine protein. Deletion of either subunit e or subunit g leads to severe disruption of the structure of the mitochondrial cristae, but neither subunit is required for the formation of an enzymatically active complex (15, 48). Like subunits e and g, subunit f is oriented with its N- and C-terminal regions in the mitochondrial matrix and the intermembrane space, respectively (37, 47, 49), but in contrast to subunits e and g, deletion of subunit f disrupted both the assembly and the activity of the complex (44). In addition to subunits ATP8, e, f, and g, the membrane domain of the bovine enzyme also contains two additional subunits, DAPIT and 6.8PL (14, 50), that have been identified also in the human enzyme (51). They are less tightly associated with the bovine complex than the other supernumerary subunits, and they require the presence of phospholipids to remain bound to the complex (14). Subunit DAPIT is encoded only in the genomes of metazoans (Fig. 6), and subunit 6.8PL is restricted to vertebrates (Fig. 7), and so neither subunit is a component of the yeast enzyme. Both have a single predicted transmembrane α-helix (Fig. 4). Conversely, the membrane domains of the yeast F-ATPase additionally contain subunits j (also called i) and k (15, 52, 53), and a recently discovered subunit l, a homologue of subunit k (54), that are unrelated in sequence to any of the bovine subunits and are not encoded in metazoan genomes. Again, each of subunits j, k, and l has a single predicted transmembrane α-helical span.

The present studies have revealed a network of cross-links between exposed lysine residues in the N- and C-terminal regions of bovine subunits e, f, g, DAPIT, and 6.8PL (see Fig. 4). Residue Lys-49 in the C-terminal region of 6.8PL is linked to the C-terminal regions subunits DAPIT, e, and f via cross-links to residues Lys-55, Lys-54, and Lys-79, respectively, and the C-terminal regions of subunits e and f are linked via a cross-link from Lys-47 in subunit e to Lys-85 in subunit f. Finally, the N-terminal regions of subunit f and g are cross-linked from Lys-15 in subunit f to Lys-65 in subunit g. This network of cross-links shows that the N-terminal regions of all five proteins are on the same side of the lipid bilayer in the intact F-ATPase and that, conversely, all of their C-terminal regions are located on the opposite side of the membrane. Although there is as yet no direct information about the orientations of the subunits in the bovine inner mitochondrial membrane, it is reasonable to assume that subunits e, f, and g have the same orientations as their yeast orthologs. In view of the cross-links between the C-terminal regions of the 6.8PL, DAPIT, and e subunits, it seems likely that 6.8PL and DAPIT also will be oriented in a similar fashion, as depicted in Fig. 4.

These studies on the supernumerary subunits of the bovine F-ATPase have little or no apparent relevance to the interpretation of the structure of the dimeric F-ATPase from the mitochondria of the alga *Polytomella* sp. at 6.5 Å resolution (55). It is known that the F-ATPase from this species and from the related alga *Chlamydomonas reinhardtii* contains the catalytic core subunits α, β, γ, δ, ϵ, oscp, a, and c plus nine “atypical” subunits (56). Atypical subunits Asa2, Asa4, and Asa7 are thought to be components of the peripheral stalk (57), which is much stouter and more elaborate than the peripheral stalk in the bovine and fungal F-ATPases. The stoichiometry of none of the atypical algal subunits is known, and there is no evident sequence relationship to any of the much more extensively studied peripheral stalk subunits in the bovine enzyme especially (6–10), and there is no relationship either of atypical subunits with any of the supernumerary subunits described here, including ATP8. Indeed, it is not clear whether or not the algal enzymes contain an ATP8 subunit (58). The information in this paper is much more likely to be helpful in establishing relatively high resolution structures of the bovine and fungal enzymes.

## Footnotes

3 The abbreviations used are:

DAPIT: diabetes-associated protein in insulin-sensitive tissues
6.8PL: 6.8 kDa proteolipid
DSS: disuccinimidyl suberate
DSG: disuccinimidyl glutarate
DSSG: di(sulfosuccinimidyl) glutarate
BS^3^: bi(sulfosuccinimidyl) suberate.
